# Mind the gap: what explains the rural-nonrural inequality in diarrhoea among under-five children in low and medium-income countries? A decomposition analysis

**DOI:** 10.1186/s12889-021-10615-0

**Published:** 2021-03-23

**Authors:** A. F. Fagbamigbe, F. F. Oyinlola, O. M. Morakinyo, A. S. Adebowale, O. S. Fagbamigbe, A. O. Uthman

**Affiliations:** 1 Department of Epidemiology and Medical Statistics, Faculty of Public Health, College of Medicine, University of Ibadan, Ibadan, Nigeria; 2 Division of Health Sciences, Populations, Evidence and Technologies Group, Warwick Medical School, University of Warwick, Coventry, UK; 3 Division of Population and Behavioural Studies, School of Medicine, University of St Andrews, Fife, UK; 4 Department of Demography and Social Statistics, Faculty of Social Sciences, Obafemi Awolowo University, Ile-Ife, Nigeria; 5 Department of Environmental Health Sciences, Faculty of Public Health, College of Medicine, University of Ibadan, Ibadan, Nigeria; 6 Techmodia, London, West Sussex UK; 7 Portsmouth Business School, Faculty of Business and Law, University of Portsmouth, Portsmouth, UK

**Keywords:** Diarrhoea, Rural-non-rural inequalities, Decomposition, Fairlie multivariable decomposition, Low- and middle-income countries

## Abstract

**Background:**

Diarrhoea poses serious health problems among under-five children (U5C) in Low-and Medium-Income Countries (LMIC) with a higher prevalence in rural areas. A gap exists in knowledge on factors driving rural-non-rural inequalities in diarrhoea development among U5C in LMIC. This study investigates the magnitude of rural-non-rural inequalities in diarrhoea and the roles of individual-level and neighbourhood-level factors in explaining these inequalities.

**Methods:**

Data of 796,150 U5C, from 63,378 neighbourhoods across 57 LMIC from the most recent Demographic and Health Survey (2010–2018) was analysed. The outcome variable was the recent experience of diarrhoea while independent variables consist of the individual- and neighbourhood-level factors. Data were analysed using multivariable Fairlie decomposition at *p* < 0.05 in Stata Version 16 while visualization was implemented in R Statistical Package.

**Results:**

Two-thirds (68.0%) of the children are from rural areas. The overall prevalence of diarrhoea was 14.2, 14.6% vs 13.4% among rural and non-rural children respectively (*p* < 0.001). From the analysis, the following 20 countries showed a statistically significant pro-rural inequalities with higher odds of diarrhoea in rural areas than in nonrural areas at 5% alpha level: Albania (OR = 1.769; *p* = 0.001), Benin (OR = 1.209; *p* = 0.002), Burundi (OR = 1.399; *p* < 0.001), Cambodia (OR = 1.201; *p* < 0.031), Cameroon (OR = 1.377; *p* < 0.001), Comoros (OR = 1.266; *p* = 0.029), Egypt (OR = 1.331; p < 0.001), Honduras (OR = 1.127; *p* = 0.027), India (OR = 1.059; *p* < 0.001), Indonesia (OR = 1.219; *p* < 0.001), Liberia (OR = 1.158; *p* = 0.017), Mali (OR = 1.240; *p* = 0.001), Myanmar (OR = 1.422; *p* = 0.004), Namibia (OR = 1.451; *p* < 0.001), Nigeria (OR = 1.492; *p* < 0.001), Rwanda (OR = 1.261; *p* = 0.010), South Africa (OR = 1.420; *p* = 0.002), Togo (OR = 1.729; p < 0.001), Uganda (OR = 1.214; p < 0.001), and Yemen (OR = 1.249; p < 0.001); and pro-non-rural inequalities in 9 countries. Variations exist in factors associated with pro-rural inequalities across the 20 countries. Overall main contributors to pro-rural inequality were neighbourhood socioeconomic status, household wealth status, media access, toilet types, maternal age and education.

**Conclusions:**

The gaps in the odds of diarrhoea among rural children than nonrural children were explained by individual-level and neighbourhood-level factors. Sustainable intervention measures that are tailored to country-specific needs could offer a better approach to closing rural-non-rural gaps in having diarrhoea among U5C in LMIC.

## Background

Diarrhoea remains a disease of major public health challenge among under-five children (U5C) across the low and middle-income countries (LMIC) [1–3]. It is a gastrointestinal infection triggered by a pathogenic microorganism that increases the frequency of watery stools for more than three or four times a day [4, 5]. It is the second leading cause of deaths among U5C across the world. Globally, diarrhoea occurs in about 1.7 billion children yearly with 525,000 deaths [6, 7]. In LMIC, U5C experience up to 5 episodes of diarrhoea in a year [4, 8] with high incidence and case-fatality ratios [9]. Specifically, more than half of the deaths caused by diarrhoea were recorded in Nigeria, India, Pakistan, Afghanistan, and Ethiopia [5].

Globally, morbidity and mortality caused by diarrhoea have reduced in advanced countries, on the contrary, it’s still a major issue of concern among the U5C in the LMIC [10, 11]. However, there was a significant decline in the number of childhood deaths due to diarrhoea from approximately 1·6 million in 1990 to 450,000 in 2016 in LMIC [12]. To achieve a further reduction in mortality to less than 1 per 1000 live births as proposed jointly by the WHO and UNICEF, it will require a context-specific grasp of the decomposition of the drivers of diarrhoea diseases at both urban and rural settings [13].

Epidemiologic studies have shown that factors that predispose children to diarrhoea are a complex interaction between socio-economic, environmental, and behavioural variables [14–17]. Diarrhoea-related morbidity and mortality among U5C have been linked to poor hygiene and sanitation practices [1, 18], limited access to improved sources of drinking water [3, 14], socioeconomic status, education level, place of residence [9], inappropriate breastfeeding [17, 18], zinc deficiency [19] and other immediate household and environmental characteristics [5, 8, 14] The higher prevalence of diarrhoea in the LMIC has been attributed to the standard of living which differs across the rural-non-rural differences in place of residence [20].

Health disparities between children living in urban and rural regions do exist due to the differences in the level of major determinants. There is ample evidence that suggests that urban areas have better health outcomes than rural areas in LMIC [2]. Consequently, the determinants of childhood diarrhoea could be country or regionally-specific based on the difference in the living environment. Household-level and neighbourhood-level factors may be important in clarifying rural-non-rural differences in diarrhoea morbidity and mortality. This, however, calls for immediate research to understanding the decomposition effect of rural-non-rural difference in the place of residence on diarrhoea episodes among U5C in LMIC.

Although the literature is replete on the risk factors of diarrhoea, there is a dearth of information on the decomposition of factors associated with the development of diarrhoea based on place of residence in LMIC. Hence, this study was designed to address this gap. Having good knowledge of the core individual-level and neighbourhood-level factors driving rural-non-rural health disparities would assist LMIC in planning appropriate intervention measures aimed at improving population health outcomes and reducing the burden of diarrhoea among U5C.

## Methods

### Study design and data

The data from the Demographic and Health Surveys (DHS) collected periodically across the LMIC was used in this study. The ICF Macro, the USA in conjunction with the ministry of health, the office of statistics, and the population commissions in respective LMIC conduct the periodical cross-sectional nationally representative population-based household DHS. We pooled data from the most recent DHS conducted within the last ten years (2010–2018) and available as of April 2020 and which provided information on diarrhoea among U5C. Only 57 LMIC met these inclusion criteria and were included in this study. We analysed data of 796,150 U5C, from 63,378 neighbourhoods across the 57 LMIC. In each of the countries, DHS used a multi-stage (usually from states/divisions/regions to the district to clusters), stratified sampling design. The households (the sampling units) are selected from the clusters known as the primary sampling units (PSU) [21, 22]. We applied sampling weights provided in the data to all our analysis. This was to adjust for unequal cluster sizes and to ensure that our findings adequately represent the target population. The DHS uses similar surveys and research protocols, standardized questionnaire, similar interviewer training, supervision, and implementation in all the countries. For full details of the sampling methodologies, please visit dhsprogram.com.

### Dependent variable

The outcome variable in this study is the recent experience of diarrhoea. Diarrhoea is defined as “passage of liquid stools three or more times a day” [4, 5] and “recent experience of diarrhoea” as having any symptom of diarrhoea within two weeks before the interview date [23]. The mothers were asked if any of their U5C had diarrhoea within two weeks preceding the survey. The responses were binary: Yes or No.

### Main determinant variable

The main determinate variable in this decomposition study is the rural-non-rural differentials in the location of the residence of the mothers. The DHS data have already classified study clusters into either rural or non-rural areas using similar standard classification procedures as of the time of the surveys with minimal differences in what rural areas were across the countries. We named children born to rural and non-rural women as rural and non-rural children respectively.

### Independent variables

The identified variables in the literature [5, 20, 24–28] and the Moseley’s systematic conceptual framework on study of child survival in developing countries was used to select the explanatory variables in this study [29]. The independent variables used in the study were based on the identified factors associated with diarrhoea among U5C in the literature [5, 20, 24–28]. These are made up of the individual-level and neighbourhood-level factors.

#### Individual-level factors

The individual-level consists of childs’ characteristics, mothers’ characteristics and the households’ characteristics. Childs’ characteristics: sex (male versus female), age in years (under 1 year and 12–59 months), weight at birth (average+, small and very small), birth interval (firstborn, < 36 months and > 36 months) and birth order (1, 2, 3 and 4+). Mothers’ characteristics: maternal education (none, primary or secondary plus), maternal age (15 to 24, 25 to 34, 35 to 49), marital status (never, currently and formerly married), employment status (working or not working). Households’ characteristics: access to media (at least one of radio, television or newspaper), sources of drinking water (improved or unimproved), toilet type (improved or unimproved), cooking fuel (clean fuel or biomass), housing materials (improved or unimproved) and household wealth index (poorest, poorer, middle, richer and richest).

#### Neighbourhood-level factors

The DHS uses “clusters” as the PSU as people of the same cluster shares similar contextual factors [21, 22]. We used the word “neighbourhood” to describe the clustering of the children within the same cluster and “neighbours” as members of the same cluster. The PSUs were identified using the most recent census in each country where DHS held. In this study, we considered neighbourhood socioeconomic status (SES) as a community-level variable. It was computed using principal component factor comprised of the proportion of respondents within the same neighbourhood without education, belonging to a household in poor wealth quintiles and unemployed.

### Statistical analyses

**Fig. 1:**
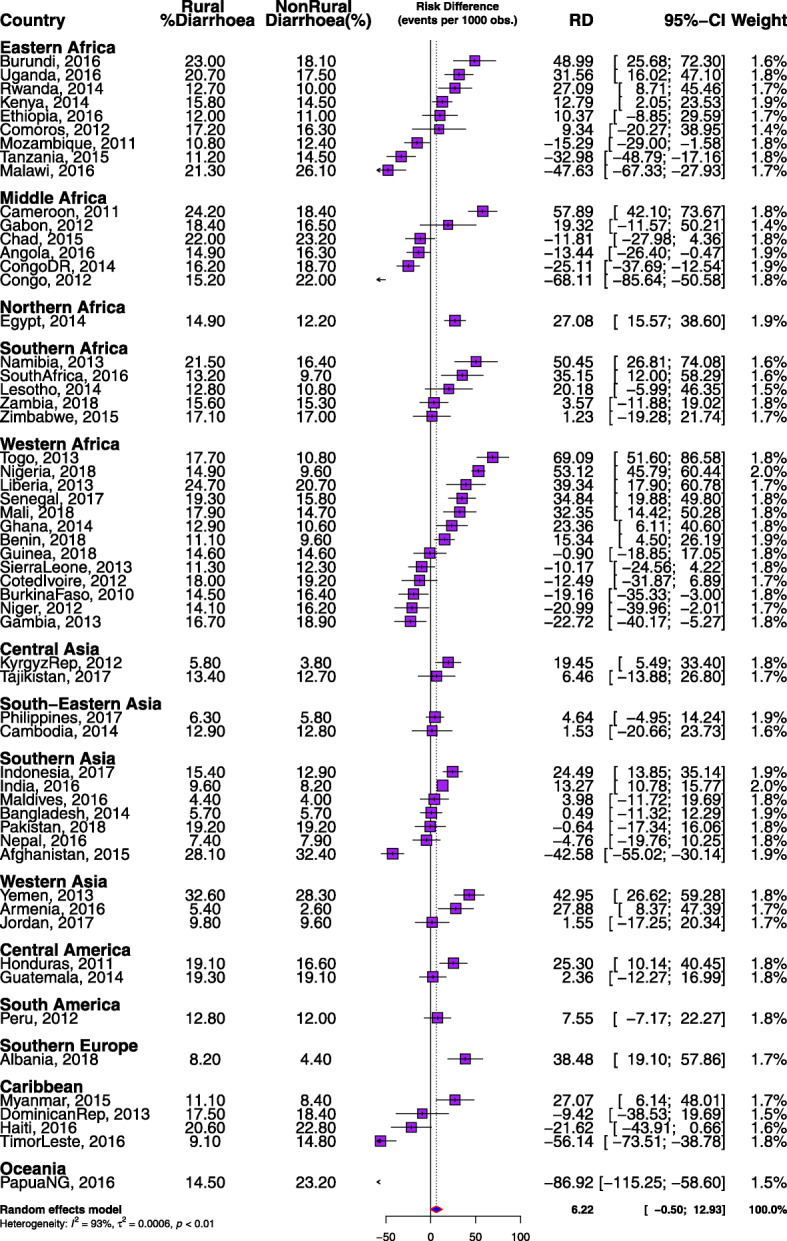
Prevalence and risk difference of diarrhoea between rural and non-rural children by countries

**Fig. 2:**
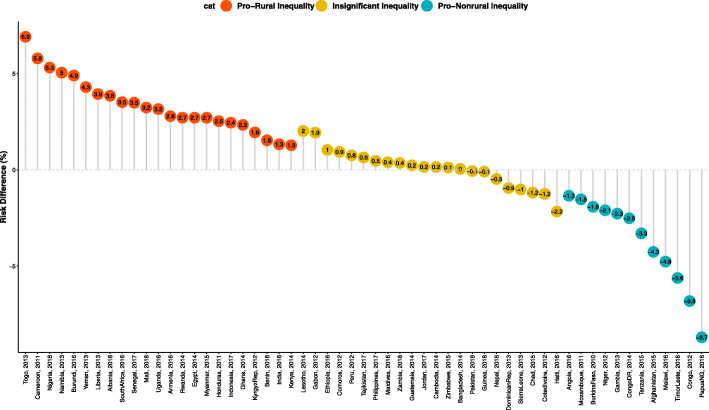
Risk difference between rural and non-rural children in the prevalence of diarrhoea by countries

**Fig. 3:**
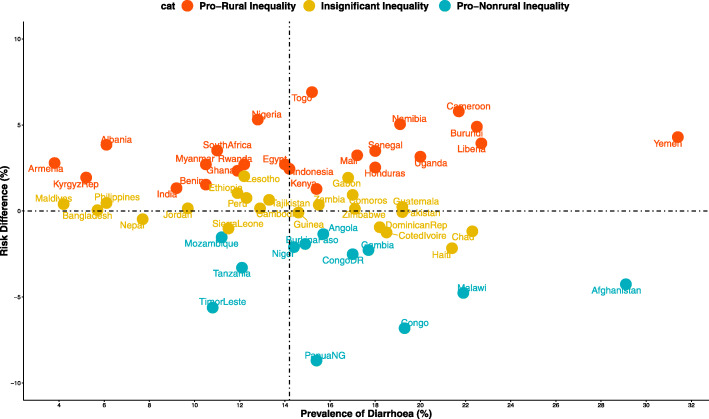
Scatter plot of prevalence and risk difference of diarrhoea between rural and non-rural children in LMIC

**Fig. 4:**
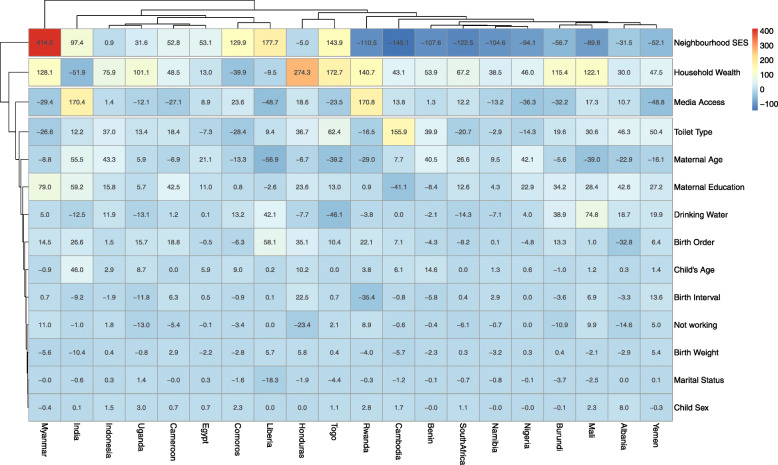
Contributions of differences in the distribution of ‘compositional effect’ of the determinants of having diarrhoea to the total gap between rural and non-rural children by countries

We used both descriptive and inferential statistics in this study. Descriptive statistics such as chats, tables, percentages were used to show the distribution of respondents by country, outcome variable and other key variables. Bivariable analysis was conducted to using the Z-test for equality of proportions who had diarrhoea among rural and non-rural children within each country and region (Table 1 (a) and (b)). We also determined the existence of an association between the explanatory variables and the outcome variable among the rural and non-rural groups of children (Table 2(a) and (b)). We carried out country-level comparison of the prevalence of diarrhoea in each of the countries by computing the risk difference (RD) in the development of diarrhoea between U5C from rural and non-rural settings and presented the results in Fig. 1. An RD greater than 0 suggests that diarrhoea is more prevalent among rural children (pro-rural inequality). Whereas, a negative RD indicates that diarrhoea is prevalent among non-rural children (pro-non-rural inequality). We estimated the fixed effects as the weighted country-specific risk differences and the random effect as the overall risk difference irrespective of a child’s country of residence. As shown in Fig. 1, forest plot was used to illustrate this distributions. Charts were used to show the distributions of the RDs (Figs. 2 and 3). We conducted tests of heterogeneity to ascertain that the 57 countries are different with regards to the odds ratio of having diarrhoea among the rural and non-rural children using adapted z-test in Stata and carried out a test of homogeneity of ORs among the 20 countries with a significant odds ratio of having diarrhoea to determine if the odds of having diarrhoea in those countries are homogenous. Lastly, the adjusted logistic regression method was applied to the pooled cross-sectional data from the 57 LMIC to carry out a Fairlie decomposition analysis (FDA) and the results presented in Fig. 4.

**Table 1:** Description of demographic and health surveys data by countries, rural percentage and diarrhoea prevalence among under-five children in LMIC, 2010–2018

Country	Year of Survey	Number of clusters	Number of Under 5 Children	Weighted (%) Rural	Weighted Diarrhoea Prevalence (%)		
					Overall	^a^Rural	^b^Non-rural
(a)							
All		63,378	796,150	68.0	**14.2	*14.6	13.4
Eastern Africa		6298	102,886	79.0	16.7	17.0	15.6
Burundi	2016	554	12,431	90.8	22.5	23.0	*18.1
Comoros	2012	252	2949	72.5	17.0	17.2	16.3
Ethiopia	2016	643	9916	88.8	11.9	12.0	11.0
Kenya	2014	1593	19,889	64.4	15.4	15.8	*14.5
Malawi	2016	850	16,246	86.8	21.9	21.3	*26.1
Mozambique	2011	610	10,157	72.4	11.2	10.8	*12.4
Rwanda	2014	492	7474	83.3	12.2	12.7	*10.0
Tanzania	2015	608	9445	73.5	12.1	11.2	*14.5
Uganda	2016	696	14,379	78.8	20.0	20.7	*17.5
Middle Africa		3081	71,630	57.7	19.0	19.2	18.8
Angola	2016	625	13,463	39.1	15.7	14.9	*16.3
Cameroon	2011	578	10,326	56.9	21.7	24.2	*18.4
Chad	2015	624	16,710	80.6	22.3	22.0	23.2
Congo	2012	384	8723	39.5	19.3	15.2	*22.0
Congo DR	2014	536	16,994	69.1	17.0	16.2	*18.8
Gabon	2012	334	5414	15.4	16.8	18.4	16.5
Northern Africa		874	15,458	68.9	14.0	14.9	12.2
Egypt	2014	874	15,458	68.9	14.0	14.9	*12.2
Southern Africa		2544	25,529	60.7	15.5	16.3	*14.3
Lesotho	2014	396	2824	72.0	12.2	12.8	10.8
Namibia	2013	536	4449	52.2	19.1	21.5	*16.4
South Africa	2016	668	3241	36.7	11.0	13.2	*9.7
Zambia	2018	545	9311	64.8	15.5	15.6	15.3
Zimbabwe	2015	399	5704	68.2	17.1	17.1	17.0
West Africa		6285	139,382	67.3	14.7	15.3	*13.4
Benin	2018	555	12,512	61.0	10.5	11.1	*9.6
Burkina Faso	2010	573	13,621	82.7	14.9	14.5	*16.4
Cote d’Ivoire	2012	351	6876	62.4	18.5	18.0	19.2
Gambia	2013	281	7633	52.7	17.8	16.7	*18.9
Ghana	2014	427	5539	55.0	11.9	12.9	*10.6
Guinea	2015	401	7213	70.4	14.6	14.6	14.6
Liberia	2013	322	6806	50.1	22.7	24.7	*20.7
Mali	2018	345	9171	79.3	17.2	17.9	*14.7
Niger	2012	476	11,437	86.3	14.4	14.1	*16.2
Nigeria	2018	1389	30,603	60.5	12.8	14.9	*9.6
Senegal	2017	400	11,253	63.1	18.0	19.3	*15.8
Sierra Leone	2013	435	10,254	74.9	11.5	11.3	12.3
Togo	2013	330	6464	63.9	15.2	17.7	*10.8
Central Asia		682	10,216	75.5	10.2	10.7	*8.5
Kyrgyz Rep	2012	316	4222	70.2	5.2	5.8	*3.8
Tajikistan	2017	366	5994	78.9	13.3	13.4	12.7
South-Eastern Asia		1850	17,168	68.3	9.0	9.8	*7.2
Cambodia	2014	609	6934	85.5	12.9	12.9	12.8
Philippines	2017	1241	10,234	55.8	6.1	6.3	5.8
(b)							
All		63,378	796,150	68.0	**14.2	*14.6	13.4
Southern Asia		33,053	322,219	70.5	11.5	11.9	*10.8
Afghanistan	2015	956	30,520	76.7	29.1	28.1	*32.4
Bangladesh	2014	600	7541	74.4	5.7	5.7	5.7
India	2016	28,321	247,181	71.6	9.2	9.6	*8.2
Indonesia	2017	1967	17,155	51.5	14.2	15.4	*12.9
Maldives	2016	265	3048	64.8	4.2	4.4	4.0
Nepal	2016	383	4827	45.9	7.7	7.4	7.9
Pakistan	2018	561	11,947	67.6	19.2	19.2	19.2
Western Asia		2048	27,441	48.8	21.8	29.1	*14.9
Armenia	2016	306	1709	42.3	3.8	5.4	*2.7
Jordan	2017	962	10,454	11.5	9.7	9.8	9.6
Yemen	2013	780	15,278	72.8	31.4	32.6	*28.3
Central America		1996	22,524	59.5	18.7	19.3	*17.8
Guatemala	2014	856	12,038	64.1	19.2	19.3	19.1
Honduras	2011	1140	10,486	53.8	18.0	19.1	*16.6
South America		1401	9408	34.3	12.3	12.8	12.1
Peru	2012	1401	9408	34.3	12.3	12.8	12.1
Southern Europe		651	2745	43.8	6.1	8.2	*4.4
Albania	2018	651	2745	43.8	6.1	8.2	*4.4
Caribbean		1860	21,129	62.8	15.0	13.6	*17.4
Dominican Rep	2013	516	3560	25.5	18.2	17.5	18.4
Haiti	2016	449	6082	64.9	21.4	20.6	*22.8
Myanmar	2014	440	4575	77.5	10.5	11.1	*8.4
Timor-Leste	2016	455	6912	71.1	10.8	9.1	*14.8
Oceania		755	8415	89.2	15.4	14.5	*23.2
Papua New Guinea	2016	755	8415	89.2	15.4	14.5	*23.2

**significant at 5% Chi-square test, *significant at 5% test of equality of proportions a and b

**Table 2:** Summary of pooled sample characteristics of the studied children in 57 LMIC

Characteristics	N	Weighted %	Weighted (%) Rural	Weighted Diarrhoea Prevalence (%)		
				Overall	Rural	Non-rural
(a)						
Age						
Infant	164,438	20.7	68.5	*17.4	*18.0	*16.1
12–59 months	631,712	79.4	67.9	13.4	13.7	12.7
Sex						
Female	389,173	48.9	68.2	*13.8	*14.1	*13.1
Male	406,977	51.1	67.9	14.6	15.1	13.7
Household Head						
Male	669,287	84.1	68.8	*14.2	14.6	*13.2
Female	126,863	15.9	64.1	14.5	14.7	14.2
Maternal age						
15–24 years	234,550	29.5	70.7	*16.4	*16.3	*16.5
25–34 years	414,014	52.0	66.3	13.2	13.7	12.3
35–49 years	147,586	18.5	68.7	13.4	14.1	12.0
Maternal Education						
No education	273,056	34.3	83.2	*15.8	*15.7	*15.9
Primary	202,835	25.5	73.9	16.3	16.3	16.3
Secondary or higher	320,257	40.2	52.2	11.7	11.7	11.7
Household Wealth Index						
Poorest	202,853	25.5	92.3	*15.1	*15.2	*13.8
Poorer	178,258	22.4	86.7	14.8	14.9	13.9
Middle	158,228	19.9	74.2	14.2	14.4	13.7
Richer	139,713	17.6	50.7	13.9	14.0	13.8
Richest	117,098	14.7	21.3	12.5	11.5	12.8
Employment						
Employed	526,983	66.2	69.4	*13.3	*13.7	*12.3
Unemployed	269,167	33.8	65.4	16.0	16.4	15.3
Media access						
No	316,993	39.9	85.0	*15.2	*15.3	*14.8
Yes	478,517	60.2	57.6	14.2	14.0	13.1
Drinking water sources						
Unimproved sources	175,663	22.8	87.0	*16.9	*16.8	*17.3
Improved sources	595,332	77.2	63.1	13.6	13.9	13.1
Toilet type						
Unimproved sources	388,386	50.4	85.3	*15.4	*15.3	*15.9
Improved sources	382,305	49.6	51.2	13.1	13.5	12.7
Marital status						
Never married	23,560	3.0	50.8	*16.9	*17.8	*15.9
Currently married	739,740	92.9	68.9	14.0	14.4	23.1
Formerly married	32,850	4.1	61.1	17.1	17.1	17.0
Cooking Fuel						
Unclean/Biomass	581,710	77.0	80.1	*14.9	*14.8	*15.1
Clean Fuel	173,921	23.0	33.3	12.4	13.3	*11.9
Housing materials						
Unimproved sources	676,227	89.5	70.9	*14.8	*15.1	*14.3
Improved source	79,157	10.5	47.2	10.0	10.1	9.9
Weight at birth						
Average+	643,472	84.0	67.4	*13.6	*14.0	*12.8
Small	90,809	11.9	70.0	17.2	17.7	16.0
Very small	31,924	4.2	70.9	20.1	20.3	19.5
Total	796,150	100.0	68.0	14.2	14.6	13.4
(b)						
Birth Interval						
1st Birth	223,779	28.2	63.0	*13.1	*13.5	*12.5
< 36 months	308,310	38.8	73.5	15.0	15.2	14.4
36+ months	262,278	33.0	66.2	14.3	14.7	13.4
Birth Order						
1st	223,777	28.1	63.0	*13.1	*13.5	*12.5
2nd	192,088	24.1	64.5	13.1	13.4	12.5
3rd	129,829	16.3	67.8	14.2	14.4	13.7
4+	250,456	31.5	75.9	16.2	*16.3	15.6
Neighbourhood SES						
Highest	159,709	20.1	37.6	*9.8	*8.8	*10.4
2	158,969	20.0	22.6	14.9	12.9	15.5
3	160,077	20.1	87.4	15.8	15.8	15.6
4	159,153	20.0	98.1	16.7	16.7	16.2
Lowest	158,242	19.9	98.2	14.0	14.1	9.9
Total	796,150	100.0	68.0	14.2	14.6	13.4

*significant at 5% Chi-square test

### Decomposition analysis

We applied Fairlie Multivariable decomposition based on the binary regression model. It belongs to the family of decomposition techniques used to quantify the contributions to differences in the prediction of an outcome of interest between two groups in multivariate models [30]. It is an extension of the Blinder-Oaxaca Decomposition Analysis (BODA) [31–33]. While the BODA works best for continuous outcomes Fairlie is renowned for the logit and probit model [34–38]. Fairlie et al. noted that the nonlinear decomposition techniques helped to overcome the challenges of the BODA when group differences are large for an independent variable [35]. We used the Fairlie methods in this study as it was purposively developed for non-linear regression models including the logit and probit models [38].

The Fairlie works by decomposing the difference in proportions based on either the probit or logit models into the portion of the characteristic [30]. The decomposition analysis was carried out by calculating the difference between the predicted probability for one group (say Group A) using the other group’s (say Group B) regression coefficients and the predicted probability for that group (Group A) using its regression coefficients [35]. The Fairlie decomposition technique works by constraining the predicted probability between 0 and 1.

Fairlie et al. showed that the decomposition for a nonlinear equation *Y* = *F*(*X*), can be expressed as:

1 $$ {\overline{\mathrm{Y}}}^A-{\overline{\mathrm{Y}}}^B=\overset{1^{st}}{\overbrace{\left[\sum \limits_{i=1}^{N^A}\frac{F\left({X}_i^A{\hat{\beta}}^A\right)}{N^A}-\sum \limits_{i=1}^{N^B}\frac{F\left({X}_i^B{\hat{\beta}}^A\right)}{N^B}\right]}}+\overset{2^{nd}\ }{\overbrace{\left[\sum \limits_{i=1}^{N^B}\frac{F\left({X}_i^B{\hat{\beta}}^A\right)}{N^B}-\sum \limits_{i=1}^{N^B}\frac{F\left({X}_i^B{\hat{\beta}}^B\right)}{N^B}\right]}} $$

Where *N*^*A*^ is the sample size for group *J* [39]. In eq. (1), $\overline{\mathrm{Y}}$ is not necessarily the same as $F\left(\overline{\mathrm{X}}\ \hat{\beta}\right)$, unlike in BODA where *F*(*X*_*i*_*β*) = *X*_*i*_*β*. The 1st term is the part of the gap in the binary outcome variable that is due to group differences in distributions of *X*, and the 2nd term is the part due to differences in the group processes determining levels of *Y* . The 2nd term also captures the portion of the binary outcome variable gap due to group differences in unmeasurable or unobserved endowments.

The estimation of the total contribution is the difference between the average values of the predicted probabilities. Using coefficient estimates from a logit regression model for a pooled sample, ${\hat{\beta}}^{\ast }$, the independent contribution of *X*_1_ and *X*_2_ to the group, gap can be written as

2 $$ \frac{1}{N^B}X\sum \limits_{i=1}^{N^B}F\left({\hat{\alpha}}^{\ast }+{X}_{1i}^A{\hat{\beta}}_1^{\ast }+{X}_{2i}^A{\hat{\beta}}_2^{\ast}\right)-F\left({\hat{\alpha}}^{\ast }+{X}_{1i}^B{\hat{\beta}}_1^{\ast }+{X}_{2i}^A{\hat{\beta}}_2^{\ast}\right) $$

and

3 $$ \frac{1}{N^B}X\sum \limits_{i=1}^{N^B}F\left({\hat{\alpha}}^{\ast }+{X}_{1i}^B{\hat{\beta}}_1^{\ast }+{X}_{2i}^A{\hat{\beta}}_2^{\ast}\right)-F\left({\hat{\alpha}}^{\ast }+{X}_{1i}^B{\hat{\beta}}_1^{\ast }+{X}_{2i}^B{\hat{\beta}}_2^{\ast}\right) $$

respectively. The contribution of each variable to the gap is thus equal to the change in the average predicted probability from replacing the group *B* distribution with the group *A* distribution of that variable while holding the distributions of the other variable constant. To obtain an accurate decomposition estimate, Fairlie et al. recommended the replication of the decomposition from a minimum of 1000 subsamples and finding the mean values of estimates from each separate decomposition [35]. Further numerical details have been reported [35, 36, 38–40]. Respectively, the contribution of each variable to the gap is thus equal to the change in the average predicted probability from replacing the group *B* distribution with the group *A* distribution of that variable while holding the distributions of the other variable constant. To obtain an accurate decomposition estimates, Fairlie et al. recommended the replication of the decomposition from a minimum of 1000 subsamples and finding the mean values of estimates from each separate decomposition [35]. Further numerical details have been reported [35, 36, 38–40].

We used the “Fairlie” command in STATA 16 (StataCorp, College Station, Texas, United States of America) to carry out the decomposition analysis to enable the quantification of how much of the gap between the “advantaged” (non-rural) and the “disadvantaged” (rural) groups is attributable to differences in specific measurable characteristics. Using the generalised structure of the model, we fitted a logistic model to determine factors influencing diarrhoea occurrence among rural and non-rural children.

## Results

### Sample characteristics and analysis of inequality

The percentage of children from rural areas was 68.0%, least (11.5%) in Jordan and the highest (90.8%) in Burundi. The overall weighted prevalence of diarrhoea was 14.2, 14.6% vs 13.4% among rural and non-rural children respectively (*p* < 0.001). The prevalence of diarrhoea among rural children ranged from 4.4% in the Maldives to 32.6% in Yemen, while it ranged from 2.7% in Armenia to 32.4% in Afghanistan among non-rural children. The z-test of equality of prevalence among the rural and non-rural children was statistically significant (*p* < 0.05) in Afghanistan (*p* < 0.001), Albania (*p* < 0.001), Angola (*p* < 0.001), Armenia (*p* < 0.001), Benin (*p* < 0.001), Burkina Faso (*p* < 0.001), Burundi (*p* < 0.001), Cameroon (*p* < 0.001), Congo (*p* < 0.001), Congo DR (*p* < 0.001), Egypt (*p* < 0.001), Gambia (*p* < 0.001), Ghana (*p* < 0.001), Haiti (*p* < 0.001), Honduras (*p* < 0.001), India (*p* < 0.001), Indonesia (*p* < 0.001), Kyrgyz Rep (*p* < 0.001), Liberia (*p* < 0.001), Malawi (*p* < 0.001), Mali (*p* < 0.001), Myanmar (*p* < 0.001), Mozambique (*p* < 0.001), Namibia (*p* < 0.001), Niger (*p* < 0.001), Nigeria (*p* < 0.001), Papua New Guinea (*p* < 0.001), Pakistan (*p* < 0.001), Rwanda (*p* < 0.001), Senegal (*p* < 0.001), South Africa (*p* < 0.001), Tanzania (*p* < 0.001), Timor-Leste (*p* < 0.001), Togo (*p* < 0.001), Uganda (*p* < 0.001), Yemen (*p* < 0.001) as shown in Table 1(a) and (b).

As shown in Table 2(a) and (b), all the explanatory variables considered were significantly associated (*p* < 0.05) with the occurrence of diarrhoea among all the children and by rural-non-rural location of residence except the sex of household head that was insignificantly associated with the occurrence of diarrhoea (*p* = 0.058) among rural children. The prevalence of diarrhoea was consistently higher among infants compared with those aged 12–59 months both in the rural area (18% vs 14%) and in the non-rural areas (16% vs 13%).

### Diarrhoea among rural and non-rural under-five children

The risk differences, a measure of inequality, in the risk of having diarrhoea among children of women from rural and non-rural areas across the countries studied are presented in Figs. 1, 2 and 3. Also, the prevalence of diarrhoea among both the rural and non-rural in each of the countries were computed and presented the results in Fig. 1. The prevalence of diarrhoea was generally higher in the rural areas than in the non-rural areas in all the countries except in Afghanistan, Angola, Burkina Faso, Chad, Congo, Congo DR, Cote d’Ivoire, Dominican Rep, Gambia, Haiti, Malawi, Mozambique, Nepal, Niger, Papua New Guinea, Sierra Leone, Tanzania, and Timor-Leste.

The pro-rural differences in diarrhoea were widest in Burundi (48.99/1000 children) and pro-non-rural RD was widest for Malawi (−47.63/1000) in Eastern Africa. In Middle Africa, the largest pro-rural difference was in Cameroon (57.89/1000) and pro-non-rural RD was highest for Congo (−68.11/1000). In The Caribbean, the pro-rural difference was widest in Myanmar (27.07/1000) and the pro-non-rural difference was widest in Timor Leste (−56.14). Irrespective of regions, the fixed effects of pro-rural differences was widest in Togo (69.09/1000) while the fixed effects of pro-non-rural differences were widest in Papua New Guinea (86.92/1000). Overall, the random effects, of the risk difference per 1000 children was 6.22/1000 children with a 95% confidence interval (CI): −0.50-12.93), evidence of insignificant overall pro-rural inequality. The greatest contribution (weight) to the random effect was found in Nigeria and India at 2% each while the least was in Comoros and Gabon at 1.4% each (Fig. 1).

In Figs. 2 and 3, we used the colours red, orange and to indicate statistically significant pro-rural inequality, insignificant inequality and statistically significant pro-non-rural inequality respectively. Based on risk differences, four of the nine countries in Eastern Africa, one of the countries in Middle Africa, Egypt in Northern Africa, two in Southern Africa and seven countries in West Africa showed statistically significant pro-rural inequality. Two countries each in Western and Southern Asia, one country each in Central Asia, Central America and Southern Europe and none in South America and Oceania had statistically significant pro-rural inequality in children having diarrhoea (Figs. 1, 2 and 3).

### Relationship between prevalence of diarrhoea and magnitude of inequality

The relationships between the prevalence of diarrhoea and the magnitude of rural-non-rural inequality, a function of RD, across the 57 countries considered in this study are presented in Fig. 3. We categorised the countries into four distinct categories based on their prevalence of diarrhoea and whether or not the differences were small or large: (i) High diarrhoea and high pro-rural inequality countries such as Togo, Yemen, Cameroun, Burundi and Namibia (ii) High diarrhoea and high pro-non-rural inequality countries such as Afghanistan, Congo, Malawi, and Papua New Guinea (iii) Low diarrhoea and high pro-rural inequality countries such as Nigeria, Egypt, South Africa and Egypt (iv) Low diarrhoea and high pro-non-rural inequality countries such as Timor Leste, Tanzania, and Mozambique.

### Decomposition of rural-non-rural inequality in the prevalence of diarrhoea

We first computed Mantel-Haenszel pooled estimate of the odds ratio (OR) of having diarrhoea while controlling for the countries among all the children. We estimated OR = 1.06 (95% CI: 1.04–1.07) and tested if the OR = 1 using z-test; and obtained z = 7.45 and *p* = 0.000 and (ii) Test of heterogeneity: *X*^*2*^ = 650.04, degree of freedom (d.f.) = 56, and p = 0.000, I-squared (variation in odds ratio (OR) attributable to heterogeneity) = 91.4%. Of the 57 countries, statistically significant pro-rural odds ratio (pro-rural inequality) was recorded in only 20 countries. The countries are Albania (OR = 1.769; *p* = 0.001), Benin (OR = 1.209; *p* = 0.002), Burundi (OR = 1.399; *p* < 0.001), Cambodia (OR = 1.201; *p* < 0.031), Cameroon (OR = 1.377; *p* < 0.001), Comoros (OR = 1.266; *p* = 0.029), Egypt (OR = 1.331; *p* < 0.001), Honduras (OR = 1.127; *p* = 0.027), India (OR = 1.059; *p* < 0.001), Indonesia (OR = 1.219; *p* < 0.001), Liberia (OR = 1.158; *p* = 0.017), Mali (OR = 1.240; *p* = 0.001), Myanmar (OR = 1.422; *p* = 0.004), Namibia (OR = 1.451; *p* < 0.001), Nigeria (OR = 1.492; *p* < 0.001), Rwanda (OR = 1.261; *p* = 0.010), South Africa (OR = 1.420; *p* = 0.002), Togo (OR = 1.729; *p* < 0.001), Uganda (OR = 1.214; *p* < 0.001), and Yemen (OR = 1.249; *p* < 0.001). All the 20 countries have statistically significant odd ratios with 95% confidence interval higher than 1 and *p*-values less than 5% alpha level as shown in Table 3. Whereas, pro-non-rural inequalities were evident in nine countries while the remaining countries experienced insignificant inequalities.

**Table 3:** The odds ratio of diarrhoea in pro-rural countries

Country	Odds Ratio	P-value	95% CI
Albania	1.769	0.002	1.232–2.538
Benin	1.209	0.002	1.075–1.360
Burundi	1.399	0.000	1.235–1.585
Cambodia	1.201	0.031	1.017–1.418
Cameroon	1.377	0.000	1.245–1.523
Comoros	1.266	0.029	1.024–1.565
Egypt	1.331	0.000	1.207–1.469
Honduras	1.127	0.027	1.013–1.253
India	1.059	0.001	1.025–1.094
Indonesia	1.219	0.000	1.119–1.329
Liberia	1.158	0.017	1.026–1.306
Mali	1.240	0.001	1.091–1.409
Myanmar	1.422	0.004	1.121–1.802
Namibia	1.451	0.000	1.240–1.697
Nigeria	1.492	0.000	1.386–1.607
Rwanda	1.261	0.010	1.056–1.506
South Africa	1.420	0.002	1.135–1.775
Togo	1.729	0.000	1.469–2.034
Uganda	1.214	0.001	1.087–1.355
Yemen	1.249	0.000	1.149–1.357

For the purpose of confirmation that the 20 countries were homogeneous as far as significant higher odds of diarrhoea among rural children than among nonrural children is concerned, we computed Mantel-Haenszel pooled estimate of the odds ratio (OR) of having diarrhoea among the children in the 20 countries while controlling for the countries. We had OR = 1.20 (95% CI: 1.17–1.23) and tested the homogeneity of the ORs: *X*^*2*^ = 144.75, degree of freedom (d.f.) = 19, and *p* = 0.000. All the tests were significant.

We included only the 20 LMIC with significantly higher odds of having diarrhoea among the rural children compared with the non-rural children in the Fairlie decomposition analysis. Figures 4 show the detailed decomposition of the part of the pro-rural inequality caused by compositional effects of the determinants of diarrhoea among under-five children by the pro-rural and pro-non-rural inequality countries respectively. The “explained” (compositional component) and the “unexplained” (structural component) portions of the rural-non-rural inequalities are depicted by red and blue colours respectively in Fig. 4. The lighter the red colour, the lower the percentage contribution of the “explained” portion and the lighter the blue colour, the lower the percentage contribution of the “unexplained” portion. We found wide variations in the factors associated with the pro-rural and pro-non-rural inequalities across the countries.

Generally, neighbourhood SES, household wealth quintile, access to media, toilet types and maternal age and education were the most important factors in most countries. Specifically, the largest contributions to pro-rural inequality in the prevalence of diarrhoea were neighbourhood SES (414% higher in communities with lowest SES), followed by household wealth index (128% higher among children from households in the poorest wealth quintiles), maternal education (79% higher among parents with no education), media access and toilet types in Myanmar. In India, the greatest contributors to the disparities are media access, neighbourhood SES, maternal education, maternal ages and birth order. The disparities were better explained by household wealth quintiles, toilet type, and maternal age in Yemen whereas the most significant contributors are neighbourhood SES, household wealth quintile, access to media, toilet types and sources of drinking water in Yemen. Other factors such as childbirth weight, age and sex, mothers’ employment status, marital status had the lowest contribution to rural-non-rural inequality in the prevalence of diarrhoea across these countries.

## Discussion

In this study, we pooled data from 57 LMIC to assess individual- and neighbourhood-level factors that explain the rural-non-rural inequalities in the development of childhood diarrhoea using the Fairlie Multivariable decomposition analysis. The study was designed on the premise that there are disparities in the health status of children living in rural non-rural areas in LMIC. We found significant disparities across countries in the factors associated with the pro-rural and pro-non-rural inequalities in the occurrence of diarrhoea. The findings in this study show the non-uniform variation in the prevalence of diarrhoea among children whose mothers reside in rural and non-rural communities. This alludes to the importance of residential inequalities in the occurrence of diarrhoea. The significant residential-related differences could be attributed to the individual characteristics across countries.

Similar to outcomes of the previous study, a higher prevalence of diarrhoea was found in the rural area as compared to non-rural areas of study. The pro-rural inequality found mostly in Asian countries as reported in the previous study [1] could be a result of a lack of social amenities and basic infrastructure needed in the rural area. In the non-rural settings, Southern and Western Asia shared the least and most burden of diarrhoea risk difference as reported in another study [41].

The study also identified factors associated with the occurrence of diarrhoea in LMIC. All the examined factors except sex of household head significantly predict the development of diarrhoea among U5C. Notably, in both rural and non-rural settings, the infants are said to be more predisposed to diarrhoea as found in the previous studies [17, 42, 43] which is said to be more pronounced among the female children though this is contrary to some studies [44, 45]. Furthermore, the age of the mother is found to be significantly associated with the development of diarrhoea as children from young mothers age 15–24 are largely exposed. This could be because at this age many of the mothers are still teenagers and some might not have the financial capability and knowledge of raising a healthy child as supported in a previous study [46]. This study also revealed that children born to non-educated mothers are susceptible to diarrhoea. Educated mothers are more likely to have adequate knowledge on the importance of good hygiene as compared to uneducated mothers. This position is supported by Fikire et al. in their study on understanding the determinants of delay in care-seeking for diarrhoea diseases among mothers/caregivers with U5C in public health facilities in Southern Ethiopia [47].

Furthermore, this study shows that unemployment [48], low economic status, and lack of access to media gadget such as television and radio among mothers are associated with the risk of their children developing diarrhoea [49]. As affirmed by other studies, an average weighted child at birth [41] firstborns [46] and children born to parents with low access to infrastructural facilities such as improved toilet [50] and improved housing materials have a higher risk of diarrhoea occurrence. Access to improved toilet facilities allows for safer disposal of faeces and limit the risk of contact between diarrhoea causative organism and human host [2].

Moreover, obvious intercountry differences in the risk-difference in diarrhoea between rural and non-rural areas were recorded. In most of the countries, the prevalence of mortality was of higher proportion in the rural areas, with an exception in 20 countries. Based on regions, the largest statistically significant pro-rural inequality in children having diarrhoea was found in Eastern Africa (in Burundi, Uganda, Rwanda and Kenya), Northern Africa (in Egypt), Southern Africa (in Namibia, South Africa). There was no significant pro-rural inequality in South-Eastern Asia and Oceania. Moreover, no significant pro-non-rural was recorded for Central Asia, Northern Africa, Southern Africa, Western Asia, Central America and Southern Europe. The inequalities observed across the countries are a pointer to the worsening health situation in the rural areas and it calls for urgent intervention.

In decomposing pro-rural inequality in the prevalence of diarrhoea in LMIC, compositional effects were found in factors such as neighbourhood SES, household wealth, wealth index, toilet type, child’s age, maternal age and contribute greatly to the prevalence of diarrhoea across countries. This invariably suggests a thorough investigation into these variables as these will go a long way in reducing Diarrhoea occurrence among children of LMIC. Specifically, in countries such as India, Yemen, and Myanmar, diarrhoea is linked to neighbourhood SES, wealth index, maternal education, and unimproved toilet types as supported by several studies [41, 51, 52]. Many of these countries are densely populated with a higher proportion of women with low socioeconomic status.

### Limitations of the study

This study has some key strengths. The use of nationally representative data generated from standardised methods in 57 LMIC gave credibility to the findings of this study in terms of generalizability across countries. The study also presented a clear pattern of diarrhoea prevalence among U5C in LMIC. One of the drawbacks in the current study was that diarrhoea morbidities were measured based on individual self-reported information which may be subject to recall bias and under-reporting and thus, distorts data quality. However, the data originators ensured the reduction of such errors during data collection. Also, the timing of data collection which vary in the studied countries may result in a bias in the comparison of diarrhoea prevalence which occurred at different periods. Besides, the cross-sectional nature of the design of the study restricts the ability to adequately establish causality and determine how rural and non-rural disparities developed over time. Moreover, the definition and categorisation of rural and non-rural areas based on certain criteria differs across countries and could limit cross-country comparisons.

## Conclusions

Our study shows significant rural-non-rural differences in diarrhoea prevalence in LMIC. The prevalence of diarrhoea was highest among children whose mothers reside in rural areas and has been linked to neighbourhood-level and individual-level factors. We found significant individual-level and community-level factors associated with pro-rural inequalities in many countries. Tackling childhood diarrhoea is not dependent on advances in technology but the adoption of interventions of proven efficacy that would further help reduce the burden of childhood diarrhoea and mortality. Sustainable intervention measures that are tailored to country-specific needs could offer a better approach to solving rural-non-rural gaps in diarrhoea prevalence in LMIC. Nonetheless, the odds of diarrhoea was higher among non-rural children in some countries. Further research is needed in this regard. However, the reasons could be ascribed to poor hygiene and sanitation in non-rural areas. More so, there are non-rural areas with slums, in which case the slums could have been categorized as non-rural areas. Of concern is also the type of food available to children in non-rural areas.

Public health and community efforts should focus on promoting hygiene programs and intervention such as hand washing, cleaning of toilet and proper disposal of waste in addition to provision of employment opportunities to women.. This calls for the formulation of effective interventions and policies that recognizes the heterogeneity of rural and non-rural communities. There is the need to formulate regional-specific policies, rather than generalised measures, in reducing the gap in rural-non-rural diarrhoea burden. Also, intervention measures that focus on the redistribution of wealth, better access to improved sanitation among others will go a long way in reducing regional inequalities in childhood diarrhoea.

## Data Availability

The data supporting this article is available at http://dhsprogram.com.

## Abbreviations

CI: Confidence Interval
DHS: Demographic and Health Survey
IRB: Institutional Review Board
LMIC: Low- And Middle-Income Countries
PSU: Primary Sample Unit
RD: Risk Difference
SES: Socioeconomic Status
U5C: Under-Five Children
UNICEF: United Nations International Children’s Emergency Fund
WHO: World Health Organization
